# Research on the Influence of Extremely Cold Environment on the Performance of Silicone Rubber and Fluorinated Silicone Rubber

**DOI:** 10.3390/polym14091898

**Published:** 2022-05-06

**Authors:** Shenghui Wang, Mengchao Hou, Kang Ma, Zhiwei Li, Hui Geng, Wenwen Zhang, Nan Li

**Affiliations:** 1 State Key Lab of Alternate Electrical Power System with Renewable Sources, North China Electric Power University, Beijing 102206, China; 13419644089@163.com (M.H.); 18810832624@163.com (K.M.); lzw13121798721@163.com (Z.L.); abc419592426@gmail.com (H.G.); zhangwenwen0710@163.com (W.Z.); 2 State Grid Baoding Electric Power Supply Company, Baoding 071000, China; gaoyawsh@ncepu.cn

**Keywords:** extremely cold environment, silicone rubber, fluorinated silicone rubber, electrical performance, mechanical performance

## Abstract

In order to study the performance variation characteristics of silicone rubber and fluorinated silicone rubber at extremely cold temperatures, two type samples were frozen for 0, 150, 300, 450, 600, 750, 900 and 1050 h in a low-temperature test chamber with a constant temperature of −50 °C. After the samples reached a certain freezing time, they were taken out and placed at room temperature for 2 h, then the breakdown voltage, mechanical tensile properties, and hardness and surface morphology were measured, and the mechanism was analyzed. The breakdown voltage, maximum tensile force, and tensile strength of the two type samples increased with freezing time. The elongation at break decreased with freezing time, but the hardness of the two materials changed little. Microcracks appeared on the surface of the samples at about 300 h and some tiny pore and holes appeared at 750 h. The length and depth of the microcracks gradually developed with freezing time. The comparative test results of the two materials showed that the performance of fluorinated silicone rubber was better than that of silicone rubber, which indicates that fluorinated silicone rubber is more stable for some applications in extremely cold environments.

## 1. Introduction

Because of its excellent electrical insulation, weather resistance, corona resistance and other properties, silicone rubber is widely used as an insulating material in power systems [1,2,3,4]. Fluorosilicone rubber material, with its stable structure and excellent performance, has been used increasingly as an electrical insulation material in harsh environment areas [5,6,7,8]. In these regions, extreme temperatures in winter can be as low as −59 °C and such low temperatures can last for a long time [9,10,11,12]. Traditional silicone rubber materials have difficulty meeting application requirements in extremely cold environments. Therefore, a study of the electrical and mechanical properties of silicone rubber and fluorosilicone rubber materials at extremely cold temperatures is of importance to guide the selection of electrical equipment materials in extremely cold areas.

Researchers have carried out relevant studies on the properties of silicone rubber and fluorosilicone rubber. Wang Hongxu et al. studied the variation in DC breakdown voltage of silicon rubber materials with different temperature and aluminum hydroxide content. It was found that the breakdown voltage of silicon rubber materials first increased and then tended to be stable with decrease in temperature [13]. Du Boxue et al. found that, in a low temperature environment, with increase in the interface pressure between the XLPE and the silicone rubber, the breakdown voltage increased [14]. Temperature and filler (ATH) level have an effect on the dielectric properties of high temperature vulcanized silicone rubber, and the dielectric strength of the material decreases with increasing temperature and ATH level [15]. The high temperature environment in the power system has a significant effect on the breakdown characteristics of silicone rubber. The electrical tree morphology is dependent on high temperature, which is related to the different movements of the molecular chain segments of silicone rubber with increasing temperature [16]. The authors of [17] researched the electrical tree in silicone with a needle-plate electrode model at different low experiment temperatures under power frequency voltage. In the initial stage, the electrical tree grew rapidly when the temperature decreased. As the temperature continued to decrease, the growth rate of the electrical tree slowed down, and the type of electrical tree also changed. E. Coser et al. found that, as the temperature of silicone rubber increased, the deformation of the sample gradually decreased, and the elastic modulus before fracture also increased [18]. Xu Qiang et al. prepared silicone rubber by blending silicone resin with different vinyl content and studied its mechanical properties. The results of a viscoelastic behavior test indicated that when the vinyl molar content in the silicone rubber was about 0.3%, the rubber showed perfect flexibility at low temperature because of its lower glass transition temperature (Tg), and the sample had a larger storage modulus and loss modulus [19]. Guo Jingang et al. found that the tensile stress–strain behavior of silicone rubber was nonlinear and highly dependent on the strain rate and temperature. The stiffness and nominal stress values at a given elongation increased with increasing strain rate and decreased with increasing temperature [20].

Based on the above research, it is evident that low temperature influences the performance of rubber materials. At present, there are few studies on the breakdown characteristics and mechanical properties of silicone rubber and fluorosilicone rubber materials under continuous extremely cold environment conditions. Based on the above research status, the effects of a continuous extremely cold environment on the breakdown voltage, maximum tensile force, tensile strength, elongation at break, hardness and surface morphology of the two materials were studied in this paper. The research results provide important guidance for the selection of suitable silicone rubber insulating materials to prevent bird damage, wind deflection, and short-circuit accidents caused by foreign objects, which result in overlap between phase and phase, or between phase and ground, in extremely cold regions.

## 2. Test Sample, Test Device and Test Method

### 2.1. Test Samples

High-temperature vulcanized silicone rubber and fluorosilicone rubber samples were provided by Hebei Gui Gu Chemical Co., Ltd. (Handan, China). The type of silicone rubber sample used was GH-10, the main components of which are methyl vinyl silicone rubber and fillers; the formula composition provided by the manufacturer is shown in Table 1. The type of fluorosilicone rubber was FG-3, the main components of which are γ-trifluoropropyl methyl vinyl silicone rubber and fillers; the formula composition provided by the manufacturer is shown in Table 2.

The production process followed by the manufacturer was as follows. The rubber mixing process was carried out in the open mill. After the raw rubber was added and rolled, then the fumed silica and structure control agent were added in batches. After all the fumed silica was mixed, iron oxide, a vulcanizing agent and other fillers were added. When the above components were mixed evenly, the mixture was thinned three times; it was then rolled and was ready to use for the next step. Vulcanization was carried out after the rubber was kept for 24 h. In the first stage the vulcanization conditions were 165 °C × 10 min × 10 MPa; in the second stage the vulcanization conditions were 200 °C × 4 h × 10 MPa.

**Figure 1:**
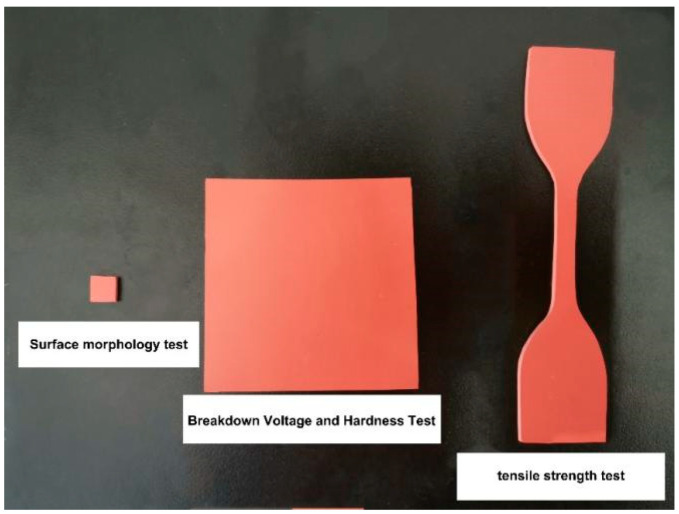
Test samples.

The preparation of test samples was performed according to the national standard DL/T376 of China. According to the relevant testing requirements, the pieces were cut into three specifications: cuboid samples of dimensions 6 mm × 6 mm × 2 mm were used for the surface topography test, larger cuboid samples of dimensions 60 mm × 60 mm × 2 mm were used for the breakdown voltage and hardness test, and specimens cut into dumbbell shapes were used for the tensile properties test. The shapes of the three samples are shown in Figure 1.

### 2.2. Test Device

#### 2.2.1. Low Temperature Test Chamber

The low temperature test chamber model used was DW-50-80L; the model has a temperature adjustable range of 0 to −50 °C, a temperature control accuracy of ±2 °C, and the temperature display is in divisions of 0.1 °C. These parameters meet the low temperature requirements of GB/T2423.1-2001.

#### 2.2.2. Power Frequency AC Voltage Withstand Test Device

The power frequency AC voltage withstand test device model used was YTC10/50J (Hubei Instrument Tiancheng Power Equipment Co., Ltd., Wuhan, China). The model has a rated voltage of AC 50 kV and a rated capacity of 10 kVA, which meet the insulation strength test requirements.

#### 2.2.3. Measuring Instrument

The tensile properties were measured by a universal testing machine (model KY-DS5Y, Shanghai Xiangjie Instrument Technology Co., Ltd., Shanghai, China). For the model, the maximum test force is 5000 N, the test precision is better than ±1%, the resolution accuracy of the beam displacement measurement is higher than 0.0025 mm, the range of test speed is 1–500 mm/min, and the speed control accuracy is better than ±1%.

The hardness was measured by a Shore hardness tester (model LX-A, Beijing Times Peak Technology Co., Ltd., Beijing, China). For the model, the test range is 100 HA, the error is ±1 HA, the pressure array stroke is 2.5 mm, the test needle is suitable for measuring rubber, synthetic rubber and other low and medium hardness materials, and the needle size is 0.79 mm.

The surface topography was measured by a G300 field emission scanning electron microscope (SEM, Carl Zeiss, Oberon Cohen, Germany). The general magnification of the SEM is 50–100,000 times and the maximum magnification is 200,000 times.

### 2.3. Test Method

#### 2.3.1. Sample Pretreatment

The silicone rubber and fluorosilicone rubber samples were divided into eight groups, with sixteen groups in total. Each group contained three different specifications of the samples, each sample having ten pieces. In order to ensure the accuracy of the experimental data, the samples were cleaned with deionized water and wiped with anhydrous ethanol. The samples were dried completely in an oven before the test.

The temperature of the low temperature test chamber was set to a constant value of −50 °C. After 24 h pre-operation, the temperature in the test chamber met the experimental requirements. The clean samples were then put into the low temperature test chamber, with freezing times of 0, 150, 300, 450, 600, 750, 900 and 1050 h. After freezing, the samples were taken out and placed for 2 h in an environment of 25 °C. When the temperature of the samples returned to room temperature, the relevant parameters were tested. In order to reduce error, the breakdown voltage and mechanical parameter values were averaged using five valid test data values.

#### 2.3.2. Breakdown Voltage

The test electrode was a rod-plate electrode. The diameter and length of the rod electrode were 1 cm and 35 cm, respectively. The rod electrode head was conical, the radius of curvature at the end was 1 mm, and the diameter of the plate electrode was 10 cm. The electrode materials were copper with good conductivity. The sample was placed on the plate electrode and the position of the rod electrode was adjusted so that its tip could just contact the upper surface of the sample. In order to prevent flashover between the rod electrode and the plate electrode along the surface of the test piece, the plate electrode and piece were placed in an organic glass barrel. Silicon oil was poured into the barrel until the liquid level just contacted the edge of the test piece; then AC high voltage was applied and the sample breakdown performance was tested.

In order to study the recovery characteristics, the breakdown voltage of the samples frozen for 300 and 450 h was tested after they were kept in a room temperature environment for 0, 3, 7 and 15 days.

#### 2.3.3. Tensile Properties

The sample was clamped symmetrically on the upper and lower grippers of the tension machine to ensure that the tension was evenly distributed on the cross section. The tensile speed was set as 500 mm/min. The testing machine was then started and continuously observed and the change in the length and force of the sample, until it was pulled off, was recorded. If the sample had not been fractured in a narrow place, the measured data were discarded.

Similarly, in order to study the recovery characteristics, the tensile parameters of the samples frozen for 300 and 450 h were tested after they had been kept in a room temperature environment for 0, 3, 7 and 15 days.

#### 2.3.4. Hardness and Surface Topography

The sample was placed on a flat and hard plane, and the pressure needle of the hardness tester was pressed vertically into the sample. The value was read within 1 s.

Before the test of surface morphology, the samples were sprayed with gold, and then the surface morphology of the sample was observed at a magnification of 500 times.

## 3. Results and Discussion

### 3.1. Influence of Low Temperature on Breakdown Voltage

**Figure 2:**
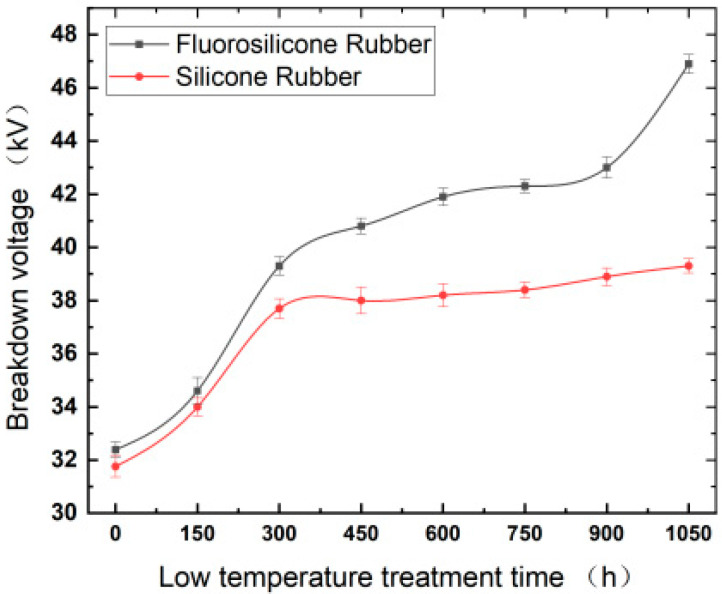
Variation of breakdown voltage with freezing time.

The relationship curves between the breakdown voltage of the samples and the cryogenic freezing time are shown in Figure 2.

By analysis of the above test results, it was found that the breakdown voltage of the silicone rubber and the fluorosilicone rubber increased greatly when the freezing time increased from 0 to 300 h. When the freezing time exceeded 300 h, the breakdown voltage of the fluorosilicone rubber increased slowly, while the voltage of the silicone rubber barely increased. After being frozen for 1050 h, the breakdown voltage of the fluorosilicone rubber increased by approximately 45% compared with the initial stage, and the value was higher than that of the silicone rubber for the whole test.

The degree of crosslinking in the silicone rubber material affects the ionization process and directly influences its breakdown characteristics. Methyl vinyl silicone rubber begins to crystallize at about −39.5 °C, and the glass transition temperature, Tg, is about −110 °C, so no glass transition occurred in our experiment [21]. Therefore, crystallization occurred inside the silicone rubber sample. In the crystalline region, small molecular chains crosslink into macromolecular chains, which inhibits the movement of free electrons. The mutual attraction between molecular chains in the crystalline region restrains the movement of disordered molecular chains in the amorphous region, resulting in reduction in the free volume, which weakens the process of electron avalanche. At the macro level, the breakdown voltage of the sample increased as the temperature decreased within the test temperature range.

Under continuous low temperature treatment, the variation trend in the breakdown voltage of silicone rubber and the fluorosilicone rubber materials is related to the intermolecular compactness, that is, the density of the raw rubber molecules relative to the aluminum hydroxide molecules [22,23]. With continuous low temperature treatment, the distribution of aluminum hydroxide molecules in the material gradually becomes denser from the surface to the inside, and the silicone rubber material molecular wraps Al(OH)_3_; this causes more aluminum hydroxide molecules to squeeze into the gaps between the various groups of methyl vinyl rubber molecules, and reduces the air permeability of the silicone rubber material making the whole material more compact inside the volume [24,25]. Therefore, the average breakdown voltage tends to increase, and the increase is relatively large at the initial stage. When the density change reaches saturation, the variation degree of breakdown characteristic of the silicone rubber material is weakened correspondingly, which indicates that the increase range is small at the macro level.

The main reason for the higher breakdown voltage of the fluorosilicone rubber is that the number of methyl groups on the surface of the fluorinated silicone rubber decreases, and the content of -CH_2_F, -CHF_2_, -CF_3_ groups in the form of covalent bonds increase. On the one hand, fluorine atoms are extremely electronegative, which can limit the delocalization of electron density. On the other hand, direct fluorination can form a fluorine-containing layer on the surface of the silicone rubber, which can inhibit the accumulation of surface charges on the insulating material and improve the electrical insulating properties of the fluorine-containing material [26].

### 3.2. Effect of Low Temperature on Breakdown Voltage Recovery Characteristics

**Figure 3:**
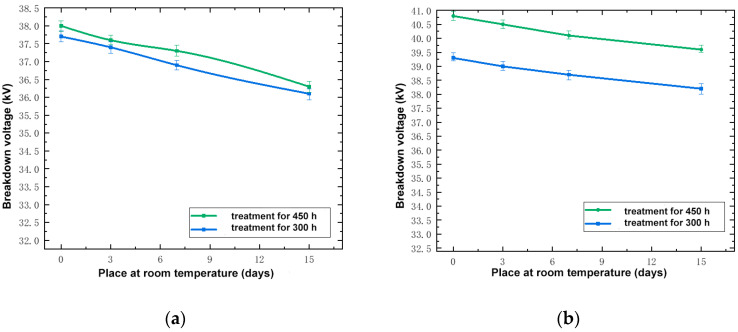
Relationship between breakdown voltage and days at room temperature. (**a**) Silicone rubber; (**b**) Fluorosilicone rubber.

In order to compare the electrical recovery performance of the silicone rubber and fluorosilicone rubber samples at room temperature, based on the test data, the relationship between the breakdown voltage of 300 and 450 h of low temperature treatment and recovery days at room temperature was determined, as shown in Figure 3.

By analyzing Figure 3, it can be seen that the longer the frozen samples were kept at room temperature, the lower their breakdown voltage, but the decreasing tendency was not marked. Taking a low temperature treatment for 300 h as an example, the breakdown voltage of the silicone rubber sample kept at room temperature for 15 days was only 1.6 kV lower than that of the sample kept at room temperature for 0 days, with a percentage decrease of 4.24%. The breakdown voltage of the fluorosilicone rubber sample kept at room temperature for 15 days was only 1.7 kV lower than that of the sample kept at room temperature for 0 days, with a percentage decrease of 4.47%. Even after being kept for 15 days at room temperature, the breakdown voltage of the two samples treated at low temperature was still much higher than that of the samples not treated; this indicated that the properties of the samples had not completely recovered to the state prior to low temperature treatment. Although the breakdown voltage of the two samples decreased, the breakdown voltage of the fluorosilicone rubber samples was still higher than that of the silicone rubber samples; this indicated that the electrical insulation performance of the fluorosilicone rubber was better than that of the silicone rubber.

During the recovery process at room temperature, the increase in ambient temperature was not conducive to the development of material crystallization, which led to a decrease in crystallinity of the sample and the gradual relaxation of the internal structure of the material. The macroscopic manifestation was that the breakdown voltage gradually decreased. That is, the low temperature treatment influenced the dielectric properties, such that the sample could not fully recover at normal temperature in a short time.

### 3.3. Effect of Low Temperature on Tensile Properties

**Figure 4:**
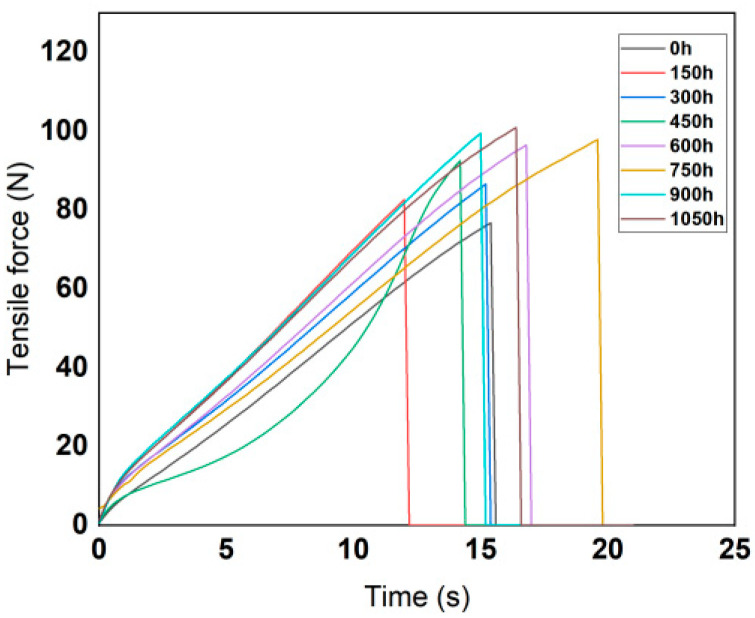
Variation curves of fluorinated silicone rubber tensile force with time.

**Figure 5:**
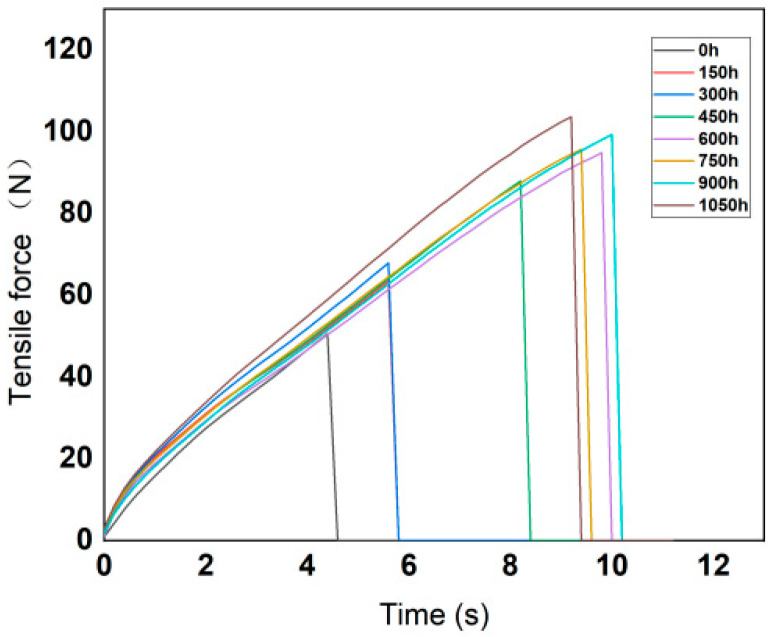
Variation curves of silicone rubber tensile force with time.

According to the technical requirements of the tensile test, the moving speed of the gripper was set to 500 mm/min. The variation curves of tensile force with tensile time are shown in the Figure 4 and Figure 5.

According to the test data, the maximum force and elongation at break of the silicone rubber and fluorosilicone rubber samples were calculated, and the results are shown in Table 3.

The tensile strength of the two kinds of rubber samples was calculated according to Formula (1):

*T_S_* = *Fm*/*Wt* (1)

*Fm*: The maximum force recorded (unit N).
*W*: The width of the narrow part of the cutter (unit mm).
*t*: The thickness of the test length (unit mm).

In the test, the dumbbell-shaped specimens were cut by a type 1 cutter; the narrowest part *W* of the cutter was 6 mm, and the thickness of the test length part of the specimen was 2 mm.

**Figure 6:**
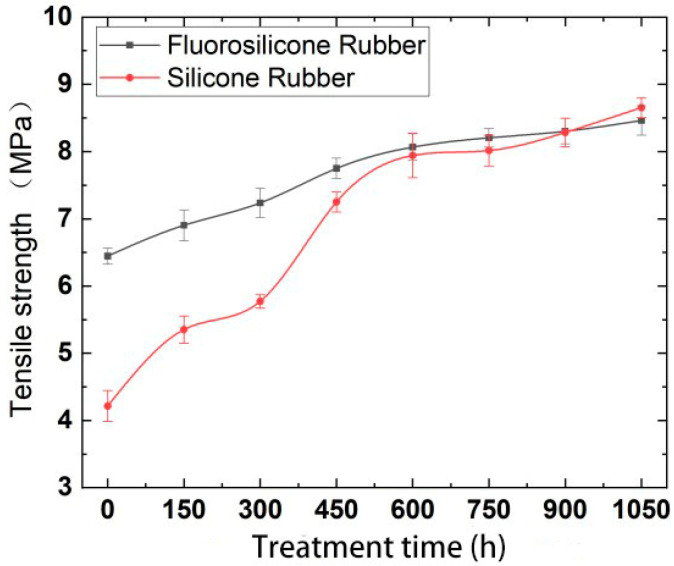
The variation of tensile strength with low temperature freezing time.

**Figure 7:**
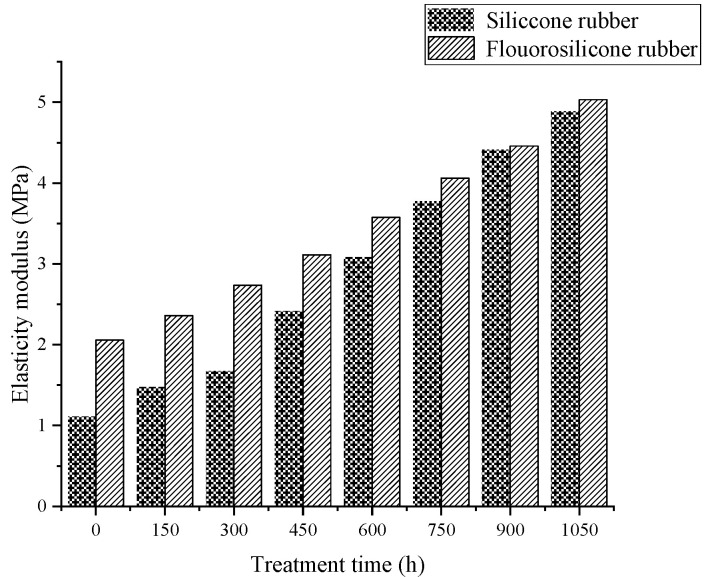
The variation of elasticity modulus with low temperature freezing time.

According to the calculation results, the variation features of tensile strength and elasticity modulus are shown in Figure 6 and Figure 7.

Analysis of the above test results showed that the elastic modulus and tensile strength of the two samples increased with increase in low-temperature freezing time. The tensile property of the fluorosilicone rubber changed smoothly and its value was almost always higher than that of the silicone rubber, which indicated that the tensile strength of the fluorosilicone rubber was greater.

After vulcanization, the rubber is crosslinked into a random network structure. In addition to the influence of intramolecular chemical bonds, tensile strength is also related to the physical crosslinking and intermolecular forces between molecules. When the rubber is stretched, the intermolecular force is first broken, and then the stress is concentrated on the oriented main chain, and physical crosslinking and chemical bond breakage occur [27].

The molecule chain structure is the main factor determining the basic properties of polymers, and the aggregation structure is the main factor determining the bulk properties of polymers. Therefore, the aggregation morphology of molecular chains has a great influence on the performance of silicone rubber, while the main factor affecting the aggregation morphology of molecular chains in silicone rubber is temperature [28]. With increase in freezing time, the thermal motion energy of polysilane molecular chains decreases, and the activity of the motion units also decreases, reflecting the volume shrinkage effect of polysiloxane molecular chains at low temperature. That is, with decrease in temperature, molecular motion activity decreases, the activity of side group and chain segments becomes more and more constrained [29], the chains intertwine more tightly, and the intermolecular force increases, so the tensile strength increases significantly, and the elongation at break decreases significantly.

Because fluorosilicone rubber is a linear polymer with Si-O-Si as the main chain structure, and fluoroalkyl or fluoroaryl groups are introduced into the side chain, it has a larger molecular weight than silicone rubber. The larger the molecular weight, the greater the van der Waals force, so that the fluorosilicone rubber chain segments do not easily slide [30], which is equivalent to the formation of physical crosslinking points between the molecules. The energy required to break the C-F bond is greater than that of the C-H bond, so the tensile properties of fluorosilicone rubber are better than that of silicone rubber after low temperature treatment.

### 3.4. Effect of Low Temperature on Tensile Properties Recovery

For comparison of the mechanical recovery properties of the frozen silicone rubber and fluorosilicone rubber samples at room temperature, the test data are shown in Table 4 and Table 5.

**Figure 8:**
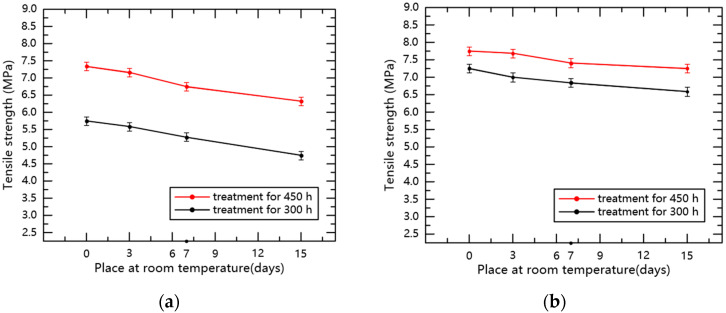
The relationship between the tensile strength of specimens and the days of storage at room temperature. (**a**) Silicone rubber; (**b**) Fluorosilicone rubber.

According to the experimental data, the relationship between the tensile strength and the days of storage at room temperature for samples with 300 and 450 h of low-temperature treatment was determined, as shown in Figure 8.

After recovering for 0 to 15 days at room temperature, the tensile properties of the silicone rubber and fluorosilicone rubber samples recovered to a certain degree. Compared with low temperature treatment for 450 h, the tensile properties of the material treated for 300 h were closer to the state before treatment, but the samples still did not completely revert to the initial state. Moreover, the tensile properties of the fluorosilicone rubber specimens were always higher than that of the silicone rubber specimens.

The main reason for the above experimental phenomenon is that the increase in temperature enhances the thermal motion of rubber molecules, and the activities of side groups and chain links intensify. The above two effects increase the distance between molecules and the volume of the sample, thereby reducing the intermolecular force. Macroscopically, the tensile characteristics will recover at room temperature, and the longer the time of low temperature treatment, the harder for the tensile characteristics of the material to recover.

### 3.5. Effect of Low Temperature on Hardness

**Figure 9:**
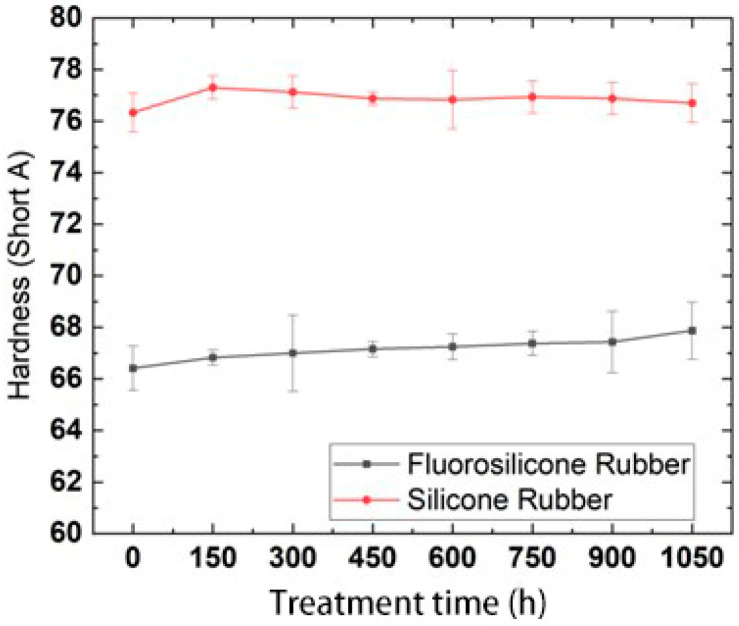
Hardness changes with low temperature freezing time.

After the samples were frozen in the low temperature test chamber for 0, 150, 300, 450, 600, 750, 900 and 1050 h, they were taken out and placed in an environment of 25 °C for 2 h to ensure that the temperature of the samples returned to room temperature. The change trend in hardness with low-temperature freezing time is shown in Figure 9.

By analysis of the above test results, it was found that the hardness of the two rubber materials showed almost no change, which indicated that the hardness values of the samples frozen at low temperature could quickly recover at room temperature. The hardness values of the fluorosilicone rubber were always lower than those of the silicone rubber, which indicated that the fluorosilicone rubber has better elasticity.

Previous studies have indicated that the polarity of the substituents in silicone rubber determines the intramolecular attraction and potential barrier, as well as the intermolecular force. The smaller the polarity of the substituent groups, the smaller the intermolecular force and potential barrier, and the more easily the molecule can rotate, so the molecule become more flexible [31]. The brittleness temperature of the two samples was about −100 °C, while the minimum temperature in the test was −50 ± 2 °C, which was much lower than the brittleness temperature. Therefore, after being kept at room temperature for 2 h, the rubber molecules could quickly recover their activity, which was not greatly different from the hardness at room temperature.

### 3.6. Effect of Low Temperature on Surface Morphology

**Figure 10:**
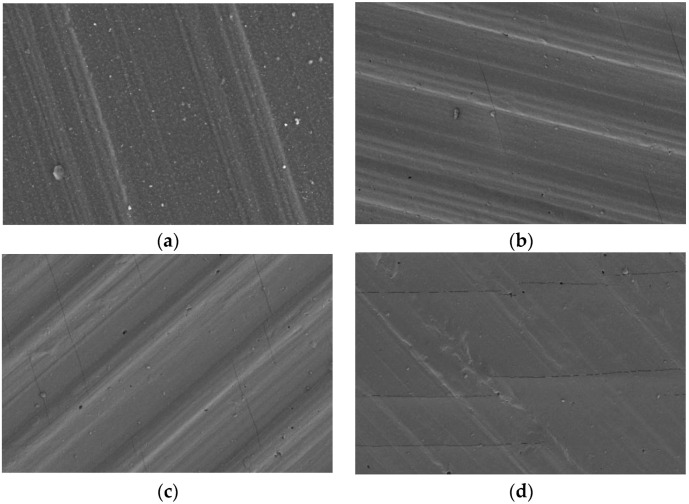
Surface morphology changes with low temperature freezing time. (**a**) Freezing 0 h, (**b**) Freezing 300 h, (**c**) Freezing 750 h, (**d**) Freezing 1050 h.

To better understand the surface state of the samples frozen at low temperature, the surface of the fluorosilicone rubber samples frozen at low temperature for 0, 300, 750 and 1050 h was observed using an SEM with a magnification of 500 times; the SEM morphology is shown in Figure 10.

By analyzing the surface topography of the above samples, it was found that the surface of the samples frozen at low temperature for 0 h remained intact. With increase in freezing time, the molecules shrink, which leads to an increase in local stress on the sample surface, with a small number of shallow cracks appearing on the sample surface after freezing for 300 h. When the freezing time increased to 750 h, the surface cracks of the samples increased and obvious holes appeared. When the freezing time reached 1050 h, the crack length and depth on the surface of the specimen markedly increased. The test results showed that freezing at extremely low temperature led to cracks and holes on the surface of the sample. However, since the breakdown voltage and tensile strength were positively correlated with the freezing time, and the depth of the SEM probe was only tens of microns, it is considered that the depth of cracks and holes on the surface of the sample was extremely small and had little influence on the electrical and mechanical properties of the sample.

Comprehensive analysis of the above test results showed that, in the extremely cold environment at −50 °C, with increase in freezing time, the performance of the fluorosilicone rubber was more stable than that of the silicone rubber. The main reason was that the C-H bond energy in the side methyl group of silicone rubber is small and easy to break, while the C-F bond energy in the side chains of fluorosilicone rubber is large and stable, so fluorosilicone rubber showed better performance [32,33]. Therefore, in order to improve the safe and stable operation of power systems, fluorosilicone rubber can be considered for electrical insulation in extremely cold areas.

## 4. Conclusions

The breakdown voltage of silicone rubber and fluorosilicone rubber increased significantly when the freezing time increased from 0 to 300 h. When the freezing time was greater than 300 h, the breakdown voltage of fluorosilicone rubber increased slowly, while the breakdown voltage of silicone rubber was basically unchanged. The breakdown voltage of fluorosilicone rubber after freezing for 1050 h was 45% higher than that of the samples without freezing, and the breakdown voltage of fluorosilicone rubber was always higher than that of silicone rubber.

After the silicone rubber and fluorosilicone rubber samples were treated at low temperature and recovered at room temperature for 0 to 15 days, the breakdown voltage of the two samples decreased, but the breakdown voltage of the fluorosilicone rubber samples was always higher than that of the silicone rubber samples; this indicated that the electrical insulation performance of the fluorosilicone rubber was better than that of the silicone rubber.

The maximum tensile force and tensile strength of the silicone rubber and the fluorosilicone rubber increased with freezing time. The tensile strength of the fluorosilicone rubber changed smoothly and its value was almost always higher than that of the silicone rubber. After the silicone rubber and fluorosilicone rubber samples treated at low temperature recovered at room temperature for 0 to 15 days, the tensile properties of the fluorosilicone rubber samples were always higher than those of the silicone rubber samples, which indicated that the fluorosilicone rubber had better tensile strength elongation performance.

The hardness of the silicone rubber and fluorosilicone rubber hardly changed with freezing time, which indicated that the hardness values of the two rubber materials can quickly recover at room temperature after freezing at low temperature. The hardness values of the fluorosilicone rubber were always lower than that for the silicone rubber, which indicated that fluorosilicone rubber had better elasticity.

There were a few shallow cracks on the surface of the specimen frozen at low temperature for 300 h. When the freezing time increased to 750 h, cracks on the surface of the specimen increased and obvious holes appeared. When the freezing time reached 1050 h, the crack length and depth on the surface of the specimen markedly increased.

## Figures and Tables

**Table 1:** Formula composition of test silicone rubber materials.

Formula Name	VQI (Mass Component Ratio)
methyl vinyl silicone rubber	100
fumed silica 4#	50–52
carbonazane	9
hydroxyl silicone oil	4
ferric oxide	1
aluminum hydroxide	110
coupling agent A151	4
2.5-dimethyl-2.5-di-tert-butyl hydroxy peroxyhexane	0.8

**Table 2:** Formula composition of test fluorosilicone silicone rubber materials.

Formula Name	VQI (Mass Component Ratio)
γ-trifluoropropyl methyl vinyl silicone rubber	100
fumed silica 4#	50–52
carbonazane	8
hydroxyl silicone oil	5
ferric oxide	1
aluminum hydroxide	110
coupling agent A151	4
2.5-dimethyl-2.5-di-tert-butyl hydroxy peroxyhexane	0.9

**Table 3:** Maximum force and elongation at break.

Freezing Time (h)	The Maximum Force of Silicone Rubber (N)	Elongation at Break (%)	The Maximum Force of Fluorosilicone Rubber (N)	Elongation at Break (%)
0	51	386	77	312
150	64	363	83	293
300	69	345	87	265
450	88	304	93	249
600	95	257	97	226
750	96	212	98	201
900	99	187	100	187
1050	104	178	102	169

**Table 4:** Data of tensile properties recovery of silicone rubber samples.

Number of Days at Room Temperature	Low Temperature Treatment for 300 h			Low Temperature Treatment for 450 h		
	The Tensile Strength (Mpa)	Elongation at Break (%)	Standard Error of Elongation at Break	The Tensile Strength (Mpa)	Elongation at Break (%)	Standard Error of Elongation at Break
0	5.75	345	0.04	7.33	304	0.04
3	5.58	355	0.06	7.17	327	0.05
7	5.33	362	0.03	6.75	348	0.05
15	4.75	370	0.07	6.33	365	0.03

**Table 5:** Data of tensile properties recovery of fluorosilicone rubber samples.

Number of Days at Room Temperature	Low Temperature Treatment for 300 h			Low Temperature Treatment for 450 h		
	The Tensile Strength (Mpa)	Elongation at Break (%)	Standard Error of Elongation at Break	The Tensile Strength (Mpa)	Elongation at Break (%)	Standard Error of Elongation at Break
0	7.25	265	0.03	7.75	249	0.05
3	7.00	277	0.06	7.67	258	0.07
7	6.83	290	0.04	7.42	274	0.03
15	6.58	307	0.05	7.25	296	0.04

## Data Availability

Not applicable.
